# Non-Hematopoietic MLKL Protects Against *Salmonella* Mucosal Infection by Enhancing Inflammasome Activation

**DOI:** 10.3389/fimmu.2018.00119

**Published:** 2018-02-02

**Authors:** Shui-Xing Yu, Wei Chen, Zhen-Zhen Liu, Feng-Hua Zhou, Shi-Qing Yan, Gui-Qiu Hu, Xiao-Xia Qin, Jie Zhang, Ke Ma, Chong-Tao Du, Jing-Min Gu, Xu-Ming Deng, Wen-Yu Han, Yong-Jun Yang

**Affiliations:** ^1^Key Laboratory of Zoonosis, Ministry of Education, College of Veterinary Medicine, Jilin University, Changchun, China

**Keywords:** mixed lineage kinase-like protein, inflammasome, gasdermin D, *Salmonella*, colitis

## Abstract

The intestinal mucosal barrier is critical for host defense against pathogens infection. Here, we demonstrate that the mixed lineage kinase-like protein (MLKL), a necroptosis effector, promotes intestinal epithelial barrier function by enhancing inflammasome activation. MLKL^−/−^ mice were more susceptible to *Salmonella* infection compared with wild-type counterparts, with higher mortality rates, increased body weight loss, exacerbated intestinal inflammation, more bacterial colonization, and severe epithelial barrier disruption. MLKL deficiency promoted early epithelial colonization of *Salmonella* prior to developing apparent intestinal pathology. Active MLKL was predominantly expressed in crypt epithelial cells, and experiments using bone marrow chimeras found that the protective effects of MLKL were dependent on its expression in non-hematopoietic cells. Intestinal mucosa of MLKL^−/−^ mice had impaired caspase-1 and gasdermin D cleavages and decreased interleukin (IL)-18 release. Moreover, administration of exogenous recombinant IL-18 rescued the phenotype of increased bacterial colonization in MLKL^−/−^ mice. Thus, our results uncover the role of MLKL in enhancing inflammasome activation in intestinal epithelial cells to inhibit early bacterial colonization.

## Introduction

The gastrointestinal (GI) tract of mammals is colonized by tens of trillions of microorganisms, which are primarily composed of bacteria, viruses, fungi, parasites, and archaea, and is constantly exposed to a wide array of these microbial antigens, requiring the mucosal immune system to induce tolerance to the commensal microbes while still mounting potent responses to pathogens (1, 2). The *Salmonella* enterica serovar typhimurium (*Salmonella*) is among the main causes of bacterial GI infections in humans and animals. According to the statistics, an estimated 20 million cases and 200,000 deaths occur due to typhoidal *Salmonella* annually (3). When *Salmonella* first enters the host it initially propagates inside the GI tract and overcomes colonization resistance provided by the gut microbiota and the innate immune system (4–6). Subsequently, to gain access to the host, it must breach the intestinal epithelial barrier (7, 8) and then translocate and/or replicate outside the gut, such as the mesenteric lymph nodes (MLNs), spleen, and liver, causing a severe inflammation of the intestinal mucosal epithelium, resulting gastroenteritis in humans, and typhoid-like systemic illness in mice (9).

As the forefront of defense against pathogens, the intestinal epithelial cells (IECs) and mononuclear phagocytes through several types of pattern recognition receptors sense both microbes and host cells that are damaged by infection, resulting in the initiation of immune responses to orchestrate a protective inflammatory response against dangerous microbes. Several families of innate receptors expressed by IECs are involved in recognition of bacterial moieties, such as Toll-like receptors (10), NOD-like receptors (11), and HIN-200 families (12, 13), which in turn help to prevent bacterial invaders and disease progression. Thus, it is not surprising that IEC-mediated innate immune signaling pathways serve a pivotal role in maintaining intestinal homeostasis.

The mixed lineage kinase-like protein (MLKL), a member of the pseudokinase family, was originally identified as an essential necroptosis effector that operates downstream of the receptor interacting protein kinase (RIPK) 1 and RIPK3 (14, 15). The activated MLKL multimerizes and translocates to the cell membrane and exposes the killer N-terminal four-helix bundle domain that results in disruption of the plasma membrane (16, 17). Since MLKL direct membrane permeabilization or pore-forming capacity was disclosed, research on MLKL has predominantly focused on its role in cell death. Evidence has shown that MLKL-mediated necroptosis participates in many inflammatory diseases (15, 18, 19) and is sufficient to protect the host against pathogens infection (20–22). However, much less is known about the biological implications of MLKL in gut including the possibility that it might elicit mucosal immunity in response to defense pathogens invasion.

In the current study, we showed that MLKL plays a critical role in intestinal defense against *Salmonella* infection and MLKL-mediated inflammasome activation in the epithelial compartment limits early bacterial mucosal colonization.

## Materials and Methods

### Mice

MLKL^−/−^ mice (C57BL/6 background, a gift from Dr. Jia-Huai Han, Xiamen University, China) (14) were backcrossed to C57BL/6 background for eight generations. C57BL/6 (WT) and MLKL^−/−^ were housed in a pathogen-free facility and have no obvious GI disorders symptoms with normal diet. The animal studies were conducted according to the experimental practices and standards approved by the Animal Welfare and Research Ethics Committee at Jilin University (No. 20150601).

### *Salmonella* Infections

Six- to eight-week-old sex-matched mice were used in this study. *Salmonella* strain SL1344 (gift from Dr. Xiang-Chao Cheng, Henan University of Science and Technology, Luoyang, China) was grown overnight at 37°C in LB broth supplemented with streptomycin (4 µg/ml) and was subsequently transferred to a high osmotic medium (a LB broth containing high concentration of sodium chloride) and incubated for 4–6 h until culture density of OD 600 reaches ~0.6. The *Salmonella*-induced colitis model was established as described previously (8). Briefly, following the administration of 20 mg of streptomycin per mouse, mice were orally inoculated with 5 × 10^7^ CFU of *Salmonella*, and 6 or 48 h p.i. Log CFU per organ was determined. For the survival study, mice were orally challenged with 1 × 10^8^ CFU of *Salmonella*.

### Evaluation of Intestinal Inflammation

Tissue samples of cecum were fixed in buffered formalin solution (4%) and embedded in paraffin. Sections (5 µm thick) were then stained with hematoxylin and eosin (H&E). *Salmonella*-induced colitis was assessed in a blinded manner using an amended version of a previously described scoring criteria (23, 24).

In brief, tissue pathology scores were determined as follows: submucosal edema (scores 0 to 3), goblet cell depletion (scores 0 to 3), epithelial integrity (scores 0 to 3), and polymorphonuclear leukocyte (PMN) infiltration (scores 0 to 4).

### Immunohistochemistry

Cecum tissue sections (5 µm) were deparaffinized and rehydrated using xylene and ethanol, respectively. Following antigen retrieval in citrate buffer (10 mM, pH = 6), samples were blocked in normal goat serum (5%) or donkey serum (5%). For immunohistochemistry, sections were stained with mucin 2 (Santa Cruz), claudin 3 (Abcam), p-MLKL (Abcam), Ly-6G/Ly-6c (BioLegend), and F4/80 (BioLegend) antibodies. Subsequently, specific staining was detected using the UltraSensitive S-P Kit and DAB Detection Kit (Maixin-Bio, China) according to the manufacturer’s directions. For immunofluorescence, tissue sections were stained with rabbit-anti-PCNA (Santa Cruz) and Alexa Fluor^®^ 488-conjugated anti-rabbit IgG (Invitrogen). Epithelial cell apoptosis was analyzed by TUNEL staining using a commercial kit (KeyGEN Biotech). DAPI (1 µg/ml) was used to stain nuclei.

### Cytokine and Chemokine Measurements

To measure the cytokine and chemokine amounts in cecum tissue, a part of cecum was homogenized mechanically in cold PBS (at a ratio of 4 ml per gram tissue) containing 1% Triton X-100 and complete protease inhibitor cocktail (Sigma-Aldrich). The mouse IL-18 ELISA kit was purchased from Arigo Biolaboratories. The other ELISA kits were purchased from R&D Systems. Cytokines and chemokines in cecum tissue were measured by ELISA according to the manufacturer’s instructions.

### Real-time PCR

RNA was isolated using TRI reagent (Sigma-Aldrich) and converted into cDNA. Subsequently, Real-time PCR assays were performed using SYBR Green (Roche) on ABI Prism 7500 sequence detection system (Applied Biosystems). Gene expression levels were calculated using the 2^−ΔCt^ method. The following primer sequences were used: GAPDH sense 5′-CACCCCAGCAAGGACACTGAGCAAG -3′, antisense 5′-GGGGGTCTGGGATGGAAATTGTGAG-3′. Occludin sense 5′-CAGCCTTCTGC TTCATCG-3′, antisense 5′-GTCGGGTTCACTCCCATTA-3′. ZO-1 sense 5′-GACCTTGAGCAGCC -GTCATA-3′, antisense 5′-CCGTAGGCGATGGTCATAGTT-3′. Claudin3 sense 5′-CCTAGGAACT -GTCCAAGCCG-3′, antisense 5′-CCCGTTTCATGGTTTGCCTG-3′.

### Immunoblotting

The ceca of mice were harvested and then homogenized in lysis buffer solution [1% Triton X-100, 50 mM Tris-HCl (pH 7.4), 150 mM NaCl, 0.1 mM Na3VO4] supplemented with complete protease inhibitor cocktail (Sigma-Aldrich). Total cell lysates were separated by SDS-PAGE and transferred to PVDF membrane. The membranes were incubated with primary antibodies against iNOS (Abcam), Cox-2 (Abcam), Claudin3 (Abcam), Caspase-1 (Santa Cruz), ASC (Santa Cruz), gasdermin D (GSDMD) (Santa Cruz), and GAPDH (Proteintech), and proteins were detected with appropriate secondary anti-rabbit or anti-mouse antibody (Santa Cruz) conjugated to horseradish and then were visualized by enhanced chemiluminescence detection reagent (Millipore).

### *In Vivo* Intestinal Permeability

An *in vivo* permeability assay was performed using FITC-dextran as described previously (25, 26). Streptomycin-pretreated WT and MLKL^−/−^ mice were orally administrated with 5 × 10^7^ CFU of *Salmonella* for 48 h. Mice were gavaged with FITC-dextran (Sigma-Aldrich) at a dose of 600 mg/kg body weight 4 h before harvest. The serum fluorescence intensity of the FITC-dextran was determined using a microplate fluorometer (Infinite M200 PRO, Tecan) with an excitation wavelength of 490 nm and an emission wavelength of 530 nm.

### Bone Marrow Chimeras

Six- to eight-week-old mice were lethally irradiated with 10 Gy of γ radiation at a rate of 1.5 Gy/min in a ^137^Cs irradiator. Within 24 h of irradiation, mice received an intravenous injection of 8 × 10^6^ bone marrow cells harvested from the femurs and tibias of WT or MLKL^−/−^ mice. The following chimeras were generated (donor bone marrow > irradiated recipient): WT > WT, WT > MLKL^−/−^, MLKL^−/−^ > MLKL^−/−^, MLKL^−/−^ > WT. Mice were allowed to recover at least 7 weeks to reconstitute the hematopoietic compartment before being used for the experiments. Efficient reconstitution by donor bone marrow cells was confirmed by PCR for the MLKL gene in splenocytes isolated from bone marrow chimeric mice (1, 27).

### Administration of Recombinant IL-18

MLKL^−/−^ mice (seven mice per group) were injected daily i.p. with PBS alone or with recombinant IL-18 (MBL International) at a dose of 1.0 µg per mouse in 100 µl PBS on days 0, 1, and 2. WT mice were injected daily i.p. with PBS alone.

### Statistical Analysis

Date are represented as mean ± SEM. Differences between mean values of normally distributed data were assessed with one-way ANOVA (Dunnett’s *t*-test) and two-tailed Student’s *t*-test. Log-rank test was used for statistical analysis of animal mortality. *p < 0.05 and **p < 0.01 compared with control group. Statistical analysis was performed using Prism (GraphPad Software, La Jolla, CA, USA).

## Results

### MLKL Plays a Critical Role in Restricting *Salmonella* Colitis

**Figure 1:**
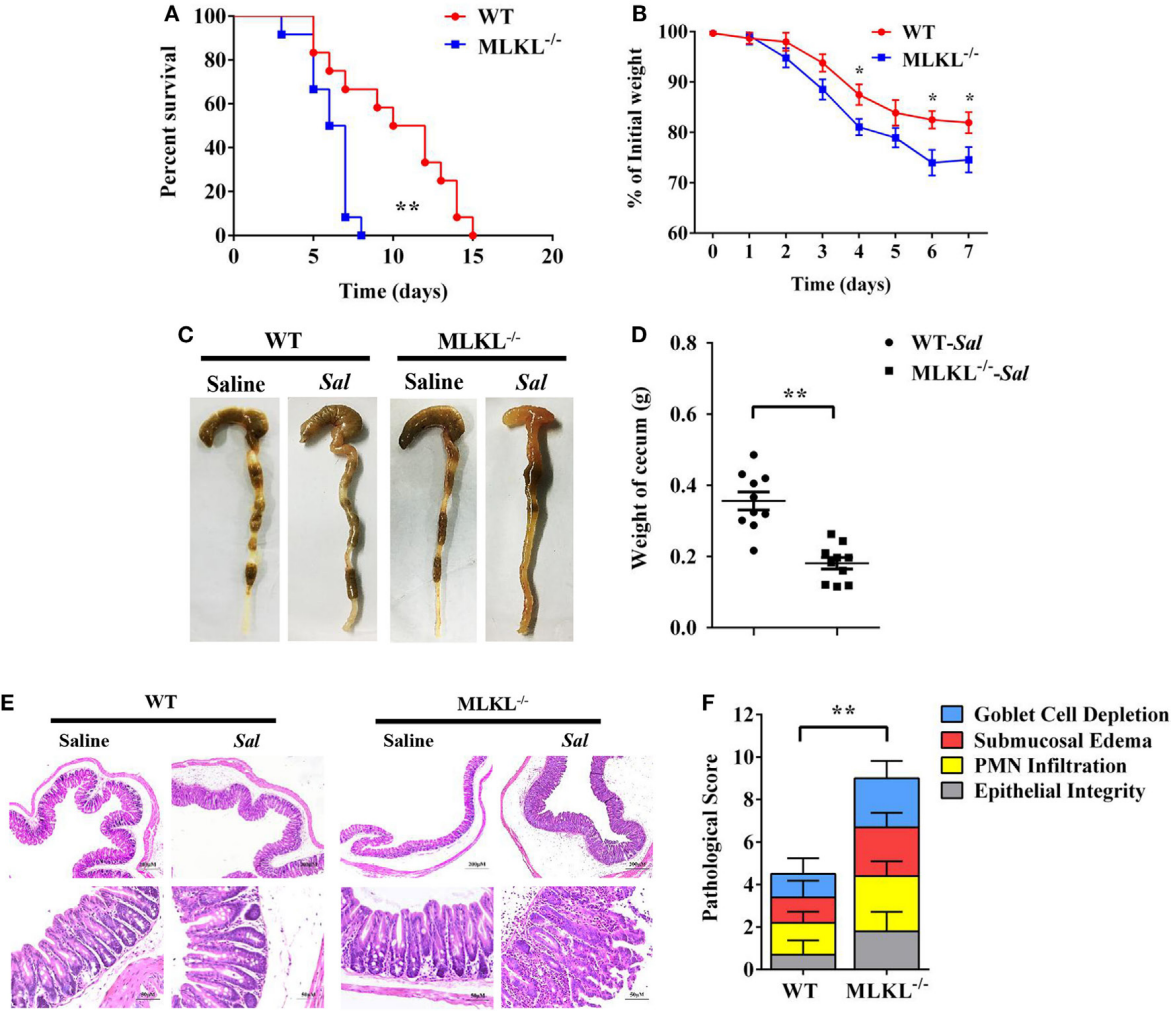
MLKL is sufficient to protect against intestinal *salmonella* infection. Streptomycin-pretreated WT and MLKL^−/−^ mice were orally infected with *Salmonella*. **(A)** Survival (1 × 10^8^ CFU, n = 12 each group). **(B)** Body weight loss (5 × 10^7^ CFU, n = 12 each group). **(C,D)** Representative gross appearance and weight of cecum (5 × 10^7^ CFU, n = 10 each group, at 48 h p.i.). **(E,F)** Representative H&E staining of cecum tissue and pathological score (5 × 10^7^ CFU, n = 10 each group, at 48 h p.i.). All data are shown as mean ± SEM. Student’s *t*-test was performed. Log-rank test was used for statistical analysis of animal mortality. Statistical significance is indicated by *p < 0.05, **p < 0.01.

To determine the biological role of MLKL in *Salmonella* infection *in vivo*, streptomycin-pretreated WT and MLKL^−/−^ mice were orally administrated with 1 × 10^8^ CFU of *Salmonella enterica subsp. enterica serovar Typhimurium* SL1344 (*Salmonella* strain SL1344), and the mortality of mice were monitored over 15 days. On day 8 p.i., we observed that all of MLKL^−/−^ mice had died, whereas almost 66.7% of WT mice remained alive, and the entire cohort of WT mice succumbed to *Salmonella* infection within 15 days (Figure 1A). The experiment was repeated with a lower infective dose of *Salmonella* (5 × 10^7^ CFU) to study the phenotype of MLKL^−/−^ mice under milder conditions. In accordance with the high level of mortality, MLKL^−/−^ mice also tended to lose more body weight and cecal weight compared to WT mice (Figures 1B–D). Simultaneously, these clinical assessments were validated by the histologic analysis of cecums. MLKL^−/−^ mice had severe intestinal damage and exacerbated intestinal inflammation, demonstrated by intense submucosal edema, goblet cell loss, massive PMN infiltration, and complete destruction of epithelial integrity (Figures 1E,F).

**Figure 2:**
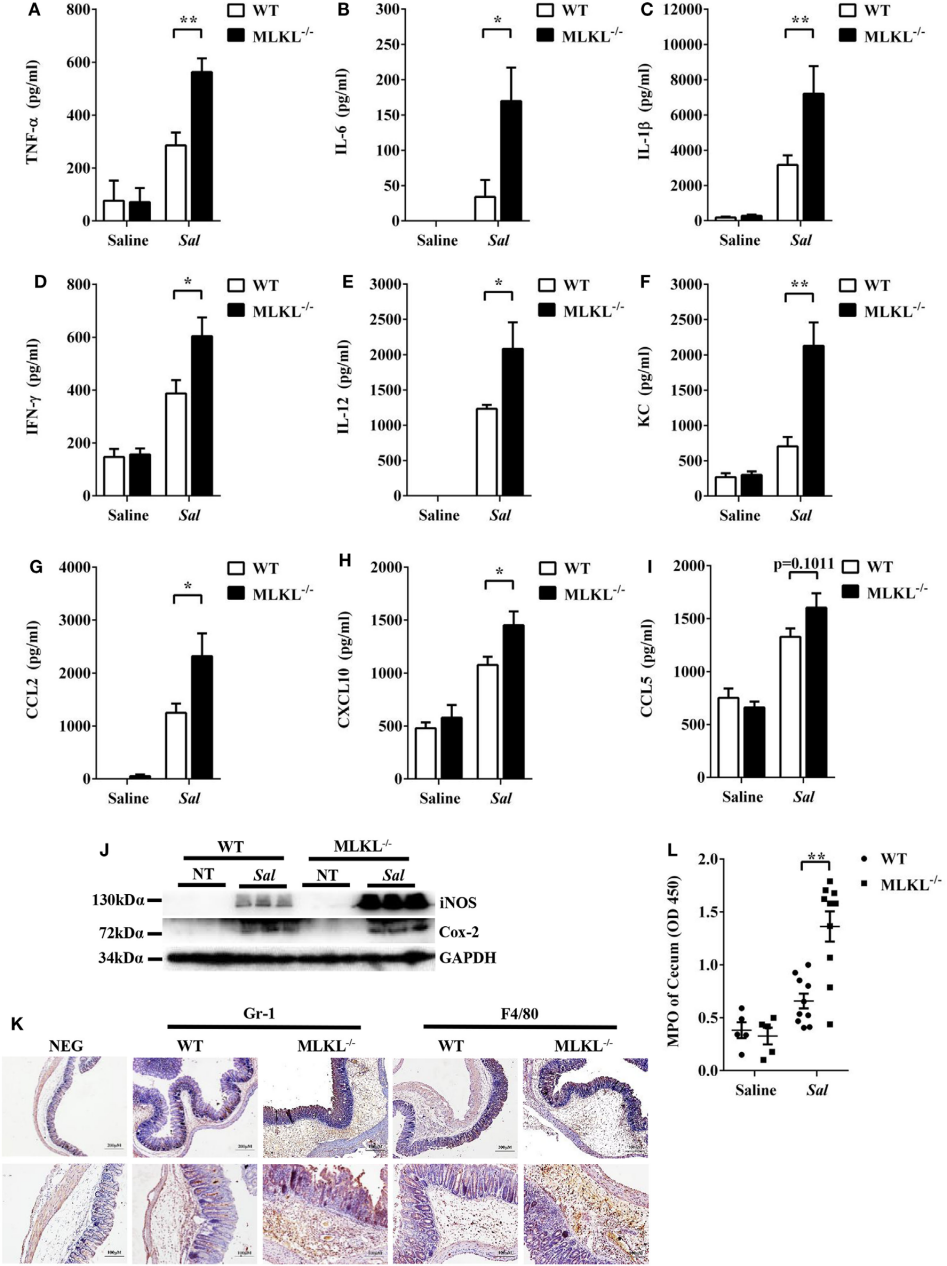
MLKL deficiency leads to enhanced pro-inflammatory mediator production following *Salmonella* intestinal infection. Streptomycin-pretreated WT and MLKL^−/−^ mice were orally infected with *Salmonella* (5 × 10^7^ CFU, *n* = 10 each group) for 48 h. The homogenate supernatant of cecum was detected for concentrations of indicated cytokines and chemokines by ELISA. **(A)** TNF-α, **(B)** IL-6, **(C)** IL-1 β, **(D)** IFN-γ, **(E)** IL-12, **(F)** KC, **(G)** CCL2, **(H)** CXCL10, **(I)** CCL5. **(J)** Representative cecum tissue lysate was analyzed for iNOS and Cox-2 by western blotting. GAPDH was used as a loading control. **(K)** Representative immunohistochemical staining of Gr-1 (a neutrophil marker) and F4/80 (a macrophagocyte marker) were performed in the cecal sections. **(L)** The homogenate supernatant of cecum was also used to determine activity of MPO (a neutrophil marker). All data are shown as mean ± SEM. Student’s *t*-test was performed. Statistical significance is indicated by *p < 0.05, **p < 0.01.

To further determine the pathologic changes in protein levels, the secreted amounts of cytokines and chemokines in cecum tissue homogenates were examined. At 48 h p.i., amounts of inflammatory cytokines (TNF-α, IL-6, IL-1β, IFN-γ, and IL-12) and chemokines (KC, CCL2, and CXCL10) were higher in the cecum tissues of MLKL^−/−^ mice than in those of WT mice (Figures 2A–H), although CCL5 secretion was comparable between both genotypes (Figure 2I). In addition, the expression of Cox-2 and iNOS, two major inflammatory mediators implicated in colorectal inflammation and cancer (28), were enhanced in MLKL^−/−^ mice (Figure 2J). Strikingly, neutrophil and macrophage accumulation were also elevated in MLKL^−/−^ mice following *Salmonella* challenge (Figures 2K,L). Conclusively, these results suggested that MLKL plays an indispensable role for protection against *Salmonella*-induced colitis.

### MLKL Deficiency Results in Increased Tissue *Salmonella* Numbers

**Figure 3:**
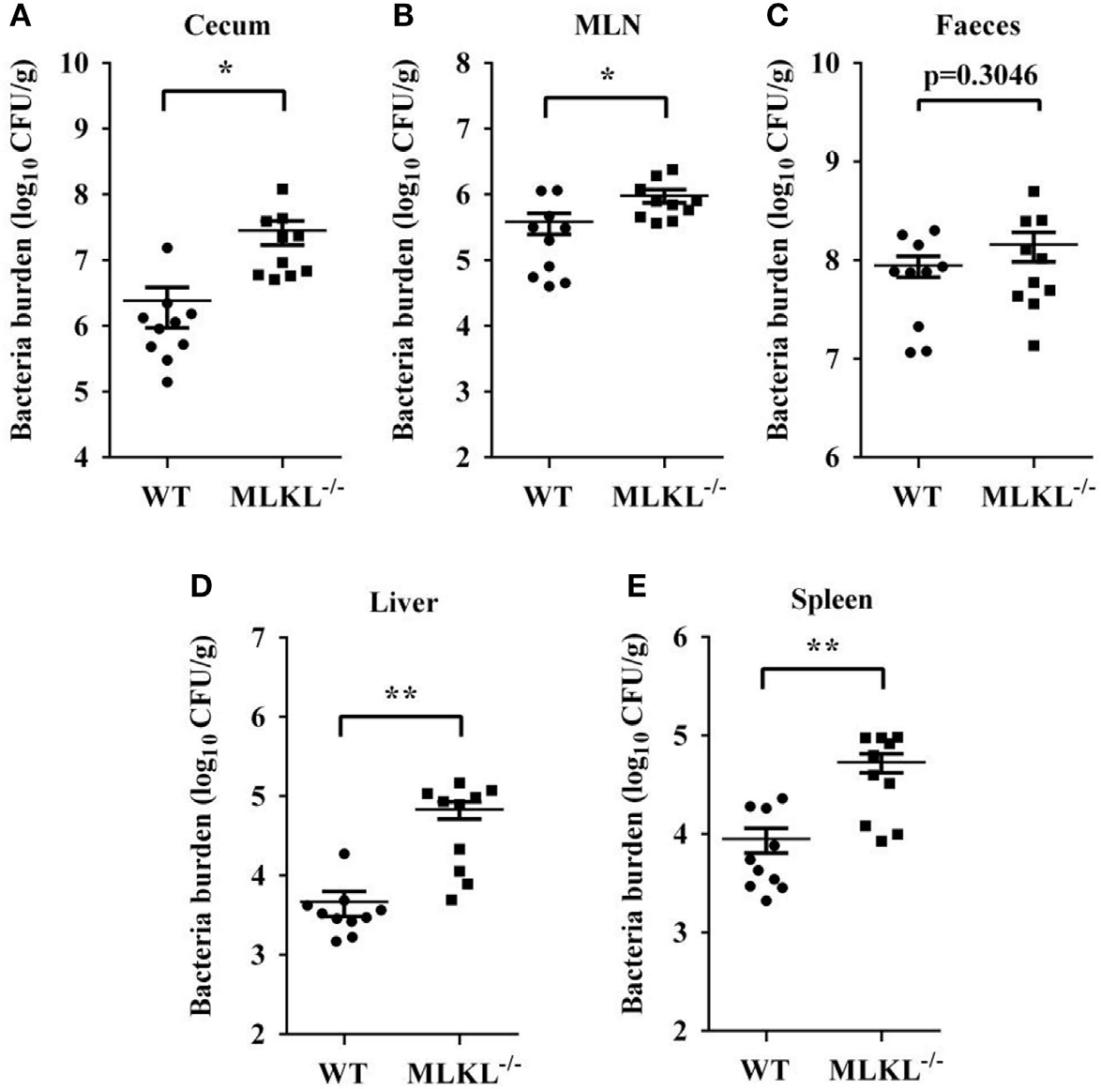
MLKL restricts *Salmonella* invasion and dissemination. Streptomycin-pretreated WT and MLKL^−/−^ mice were orally infected with *Salmonella* (5 × 10^7^ CFU, n = 10 each group) for 48 h. Bacterial load in the cecum **(A)**, mesenteric lymph node (MLN) **(B)**, feces **(C)**, liver **(D)**, and spleen **(E)** were detected. All data are shown as mean ± SEM. Student’s *t*-test was performed. Statistical significance is indicated by *p < 0.05, **p < 0.01.

Based on the above results, we speculated that increased pathology in MLKL^−/−^ mice during *Salmonella* infection might link to increased colonization levels. Whereas recovery of *Salmonella* from feces of MLKL^−/−^ mice did not differ from that from feces of WT mice, MLKL^−/−^ mice harbored significantly elevated loads of *Salmonella* in the cecum and the MLN (Figures 3A–C), indicating that MLKL plays a protective role against intestinal translocation of *Salmonella*. Moreover, the *Salmonella* CFU were strongly increased in the liver and spleen of MLKL^−/−^ mice compared to WT mice (Figures 3D,E), indicating that MLKL^−/−^ mice are more susceptible to dissemination of *Salmonella*. Thus, these results suggested that MLKL-mediated protection against mucosal *Salmonella* infection may depend on the limitation of initial intestinal invasion.

### MLKL Alleviates the Disruption of Intestinal Mucosal Barrier Integrity

**Figure 4:**
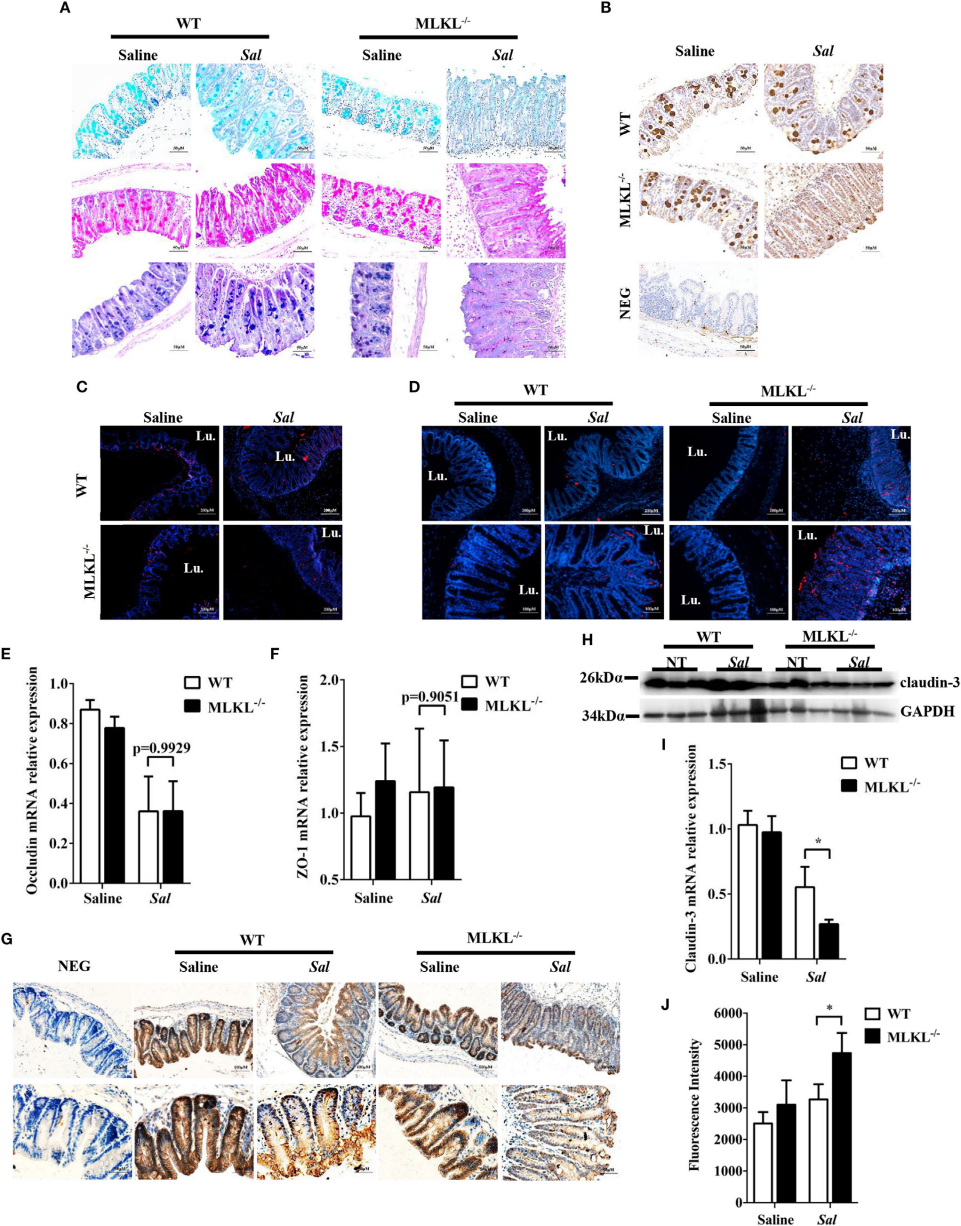
MLKL deficiency leads to severe disruption of intestinal mucosal barrier following *Salmonella* intestinal infection. Streptomycin-pretreated WT and MLKL^−/−^ mice were orally infected with *Salmonella* (5 × 10^7^ CFU) (n = 10 each group) for 48 h. **(A)** AB-PAS staining. **(B)** Mucin 2 (IHC) stained brown. **(C,D)** PCNA and TUNEL staining of proliferating cells and apoptotic cells in the cecum tissue, respectively. The cecum tissue mRNA was examined for Occludin **(E)** and ZO-1 **(F)** and claudin 3 **(I)** by QRT-PCR. The data were normalized to GAPDH expression and are showed as the fold increase in mRNA. **(G)** Claudin-3 (IHC) stained brown. **(H)** The cecum tissue lysate was analyzed for claudin-3 by western blotting. GAPDH was used as a loading control. **(J)** The intestinal permeability was measured using FITC-dextran as described in Section “Materials and Methods” (n = 5–7 each group). All data are shown as mean ± SEM. Student’s *t-*test was performed. Statistical significance is indicated by *p < 0.05, **p < 0.01.

Next, we sought the cause of the heightened bacteria load seen in *Salmonella*-infected MLKL^−/−^ mice. The intestinal epithelia barrier, primarily made up of the mucus gel layer covering the epithelium, IECs, and tight junctions, can effectively prevent enteric bacterial pathogens from penetrating the intestinal mucosa and invading into deep tissues (29, 30). We first investigate whether MLKL deficiency affects mucin secretion and glycosylation patterns. At 48 h p.i., cecal tissues were collected and stained them with alcian blue as well as periodic acid–Schiff’s reagent (AB-PAS staining). Indisputably, AB-PAS stains acidic carbohydrates blue and neutral carbohydrates pink or magenta, while tissues containing both acidic and neutral mucins are stained dark blue or purple. In WT mice, the amount of mucoprotein expression was relatively larger in *Salmonella-*infected mice than that of uninfected mice. Compared with WT mice, the AB-PAS staining mucins were dramatically reduced in MLKL^−/−^ mice after infection (Figure 4A). Mucin 2, the major component of the mucus layer, also trended to decrease in MLKL^−/−^ mice following *Salmonella* infection (Figure 4B). Thus, these data imply that mucosal infection leads to deregulation of the expression of mucoproteins in MLKL^−/−^ mice, potentially as a pathogenic mechanism in *Salmonella* colitis.

Subsequently, we examined the impact of MLKL deficiency on tissue homeostasis by examining the epithelial cell proliferation and cell death. Although there was no obvious difference in the PCNA-positive cells between WT and MLKL^−/−^ epithelial crypts (Figure 4C), TUNEL staining of histological sections of cecum showed that MLKL^−/−^ mice had greatly increased numbers of TUNEL-positive cells in the epithelium and lamina propria than in those of WT mice (Figure 4D), indicating that MLKL^−/−^ mice have a defect in maintaining enterocyte proliferative/apoptotic homeostasis following mucosal *Salmonella* challenge.

As intercellular junctions are crucial for epithelial barrier function, we wondered if *Salmonella* infection also induced any changes in tight junctions between WT mice and MLKL^−/−^ mice. The tight junction, composed of claudin, occludin, and zonula occludens (ZO)-1, has been shown previously to protect against *Salmonella* infection (8). Despite there were no difference in the expression levels of occludin and ZO-1 (Figures 4E,F), the expression level of claudin-3, a major sealing junctional component for early noninvasive detection of intestinal tight junction integrity loss (31), was significant lower in MLKL^−/−^ mice than in WT mice (Figures 4G–I). Subsequently, to further determine whether MLKL protection against *Salmonella* was associated with preservation of intestinal barrier function, paracellular intestinal permeability was assessed by oral administration of the fluorescent tracer FITC-dextran. As expected, fluorescence intensity of serum showed that MLKL^−/−^ mice had the highest levels of FITC-dextran in the blood than in those of WT mice (Figure 4J), suggesting that MLKL^−/−^ mice exhibit a disruption of barrier function. Conclusively, these results indicated that MLKL deficiency results in increased intestinal barrier damage following mucosal *Salmonella* infection.

### MLKL Potentially Limits Early Intestinal Bacterial Colonization

**Figure 5:**
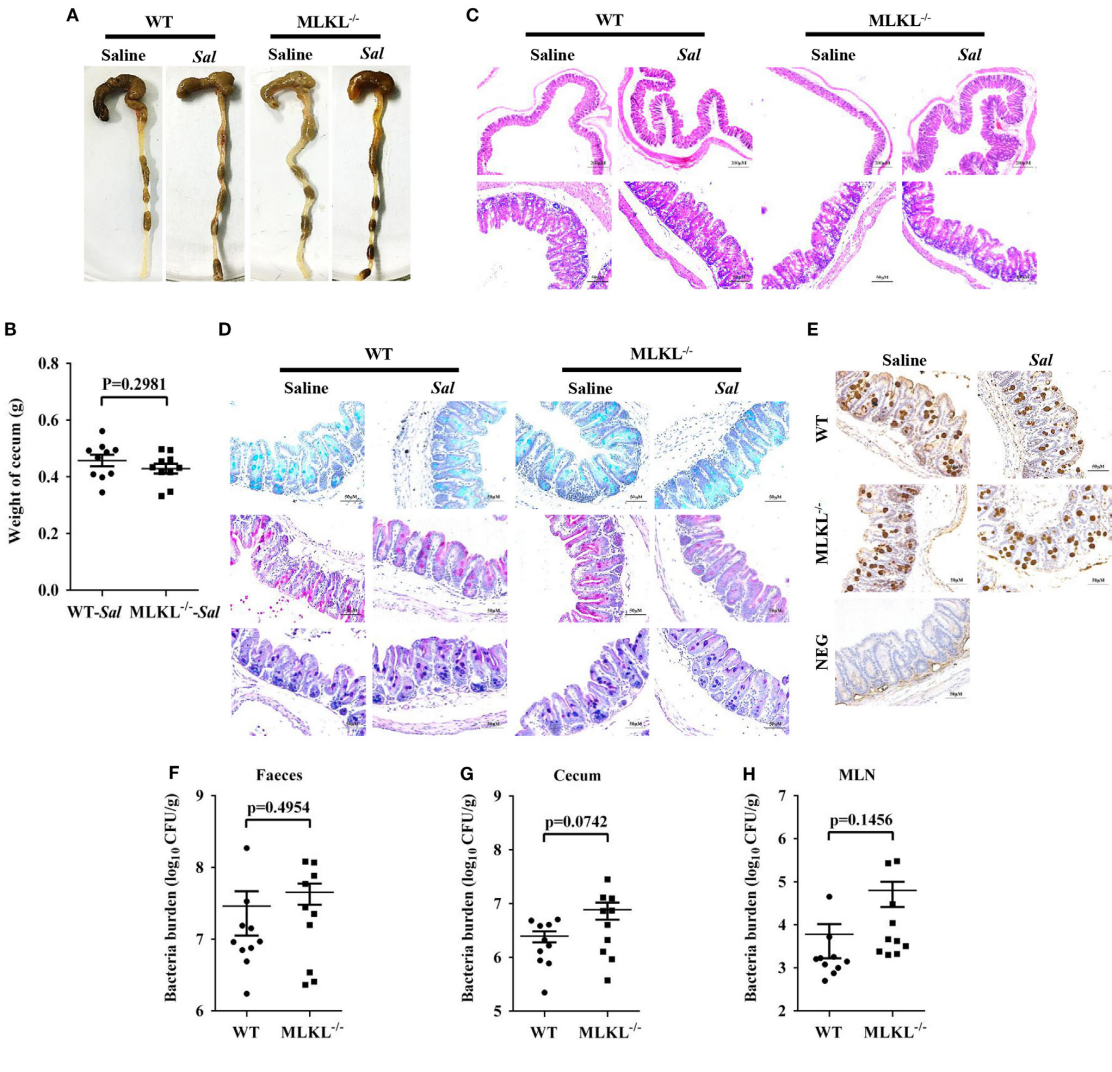
MLKL effectively restricts the acute *Salmonella* mucosal infection. Streptomycin-pretreated WT and MLKL^−/−^ mice were orally infected with *Salmonella* (5 × 10^7^ CFU) (n = 10 each group) for 6 h. **(A,B)** Gross appearance and weight of cecum. **(C)** Representative H&E staining of cecum tissue. **(D)** AB-PAS staining. **(E)** Mucin 2 (IHC) stained brown. **(F–H)** Bacterial load in the feces, cecum, and MLN. All data are shown as mean ± SEM. Student’s *t*-test was performed. Statistical significance is indicated by *p < 0.05.

The exaggerated loss of the intestinal mucosal integrity in MLKL^−/−^ mice on 48 h p.i. may be the result of exacerbated inflammation or increased bacteria localization. Therefore, to further investigate the mechanisms for MLKL mediates protection, we analyzed the initial interplay between bacteria and the host at 6 h after gavage with 5 × 10^7^ CFU *Salmonella*, since the cecal lumen was fully colonized by 2–6 h p.i (32). The clinical and histologic assessments of the infected MLKL^−/−^ and WT mice showed no obvious change compared with the controls (Figures 5A–C). Simultaneously, mucins secretion in MLKL^−/−^ mice was not different from that in WT mice before and after infection (Figures 5D,E). Subsequently, we enumerated *Salmonella* in feces, cecum, MLN, liver, and spleen. Bacteria loads in feces of both WT and MLKL^−/−^ mice were comparable at 6 h p.i. (*p* = 0.4954, Figure 5F), like those at 48 h p.i. Although no viable bacteria were detected in the liver and spleen of mice at this early stage of infection, we noted that a 0.5–1 Log increase in bacterial numbers was detected in cecum (*p* = 0.0742) and MLN tissues (*p* = 0.1456) of MLKL^−/−^ mice compared with WT mice (Figures 5G,H). These data suggest that the potential capacity of limiting early intestinal colonization underlies the protection role of MLKL, and the compromised colonization defense of MLKL^−/−^ mice leads to severe intestinal inflammation and barrier integrity disruption.

### MLKL Expression in Non-Hematopoietic Cells Mediates Protection against Infection

**Figure 6:**
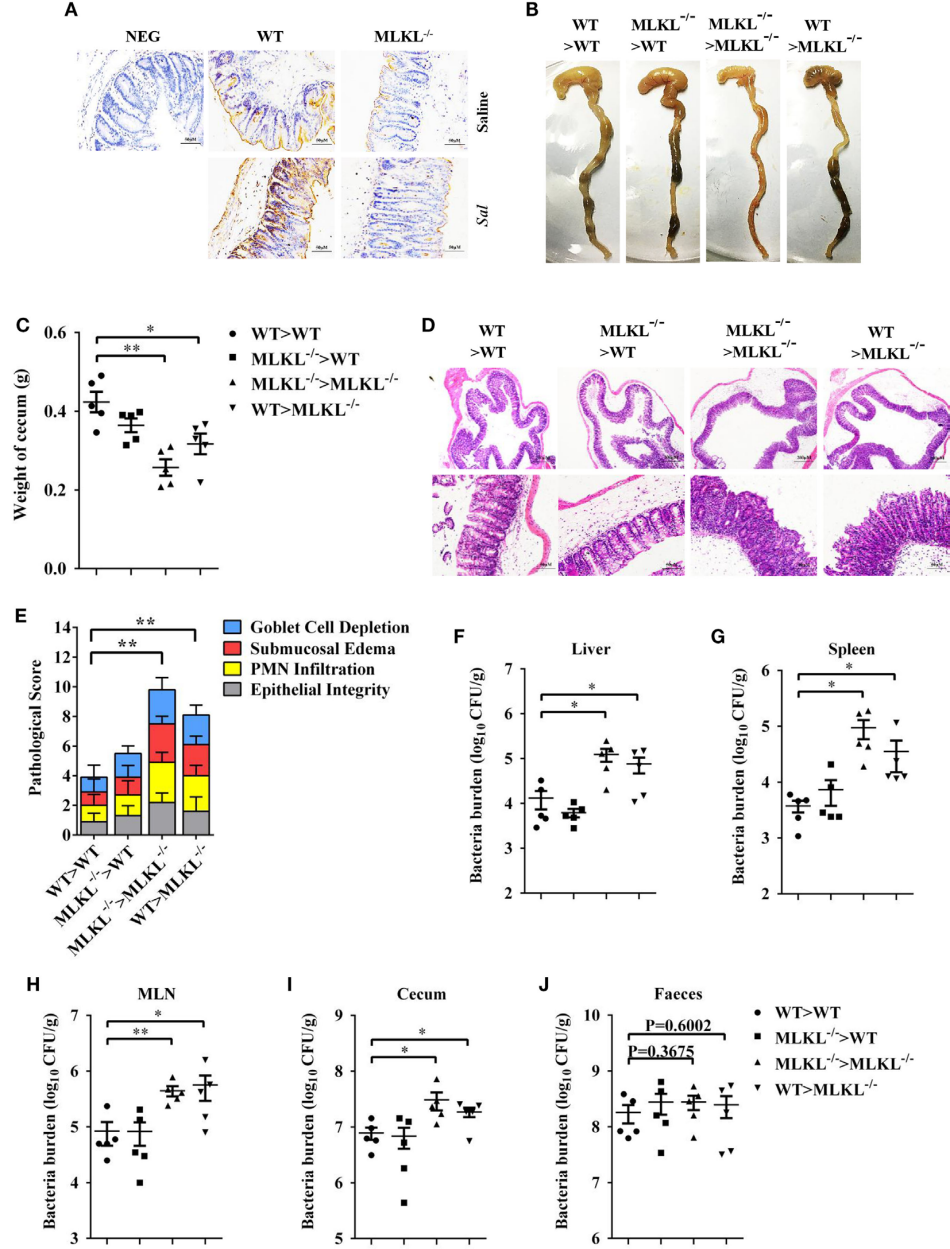
MLKL expression in non-hematopoietic cells contributes to protection during *Salmonella* infection. Streptomycin-pretreated WT and MLKL^−/−^ mice were orally infected with *Salmonella* (5 × 10^7^ CFU, n = 10 each group) for 48 h. **(A)** Representative cecum sections. Phosphorylated MLKL (p-MLKL IHC) stained brown. **(B–J)** MLKL^−/−^ and WT bone marrow chimeric mice were generated as described in Section “Materials and Methods.” Streptomycin-pretreated mice were infected orally with *Salmonella* (5 × 10^7^ CFU, n = 5 each group). **(B,C)** Gross appearance and weight of cecum. **(D,E)** Representative H&E staining of cecum tissue and pathological score, and bacteria numbers in livers **(F)**, spleens **(G)**, MLN **(H)**, cecum **(I)**, and feces **(J)** were determined. All data are shown as mean ± SEM. Student’s *t*-test was performed. Statistical significance is indicated by *p < 0.05, **p < 0.01.

To further characterize the role of MLKL in defense against intestinal colonization with *Salmonella*, the expression of phosphorylated MLKL (p-MLKL) in the cecal tissues of infected WT mice was investigated by immunohistochemical staining. Our results showed that p-MLKL was located in inflammatory cells which infiltrated to the submucosa, but predominantly expressed in crypt epithelial cells (Figure 6A). To determine in which cell populations MLKL expression mediated protective effects against *Salmonella* infection, we generated bone marrow chimeras. Thus, WT or MLKL^−/−^ recipient mice were lethally irradiated and injected with bone marrow cells from WT or MLKL^−/−^ donors. After hematopoietic reconstitution, these mice were infected with *Salmonella*. Similar to complete MLKL-deficient chimeras (MLKL^−/−^ donor > MLKL^−/−^ recipient), chimeras selectively deficient in MLKL expression in the non-hematopoietic cells (WT > MLKL^−/−^) were susceptible to oral *Salmonella* infection, as seen by severe histopathology. Conversely, chimeras in which MLKL was selective deficient in hematopoietic cells (MLKL^−/−^ > WT) were resistant to oral *Salmonella* infection, as seen by minimal histopathology identical to that observed in control chimeras (WT > WT) (Figures 6B–E). The same pattern was observed with respect to bacterial localization, with chimeras deficient in MLKL in the non-hematopoietic cells (MLKL^−/−^ > MLKL^−/−^ and WT > MLKL^−/−^) having higher bacterial burden in liver, spleen, MLN, cecum, and feces compared to chimeras with a WT non-hematopoietic compartment (WT > WT and MLKL^−/−^ > WT) (Figures 6F–J). Collectively, these data suggest that MLKL expression in epithelial cell mediates host defense against *Salmonella* mucosal invasion.

### MLKL-Mediated Inflammasome Activation Limits Bacterial Cololization

**Figure 7:**
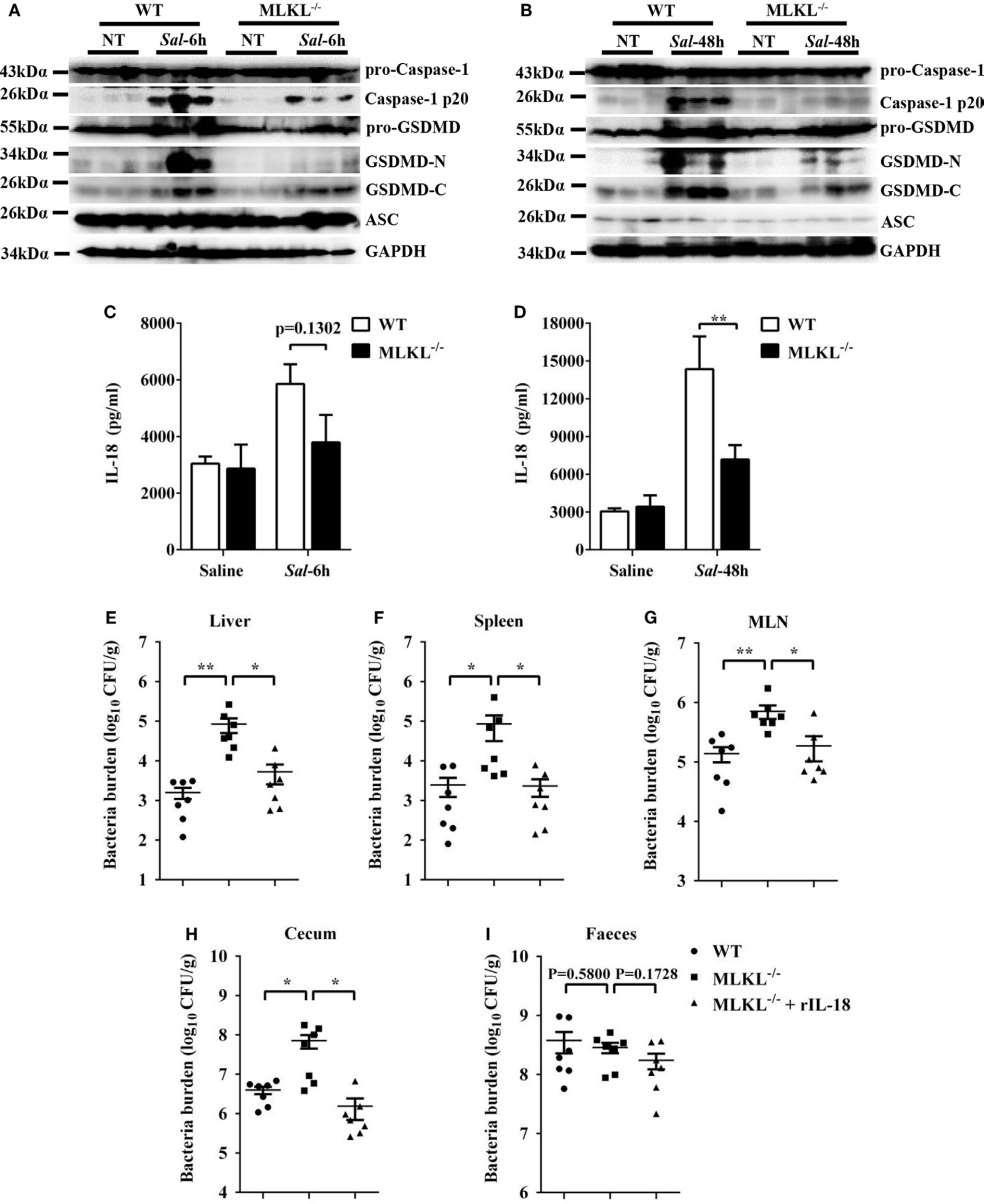
Inflammasome signaling downstream of MLKL confers protection against *Salmonella* infection. Streptomycin-pretreated WT and MLKL^−/−^ mice were orally infected with *Salmonella* (5 × 10^7^ CFU, n = 10 each group) for 6 or 48 h. **(A,B)** The cecum tissue lysate was analyzed for caspase-1, GSDMD, and ASC by western blotting. GAPDH was used as a loading control. **(C,D)** The homogenate supernatant of the cecum tissue was analyzed for the presence of IL-18 protein using ELISA. For one group of MLKL^−/−^ mice, 1.0 µg recombinant IL-18 was injected intraperitoneally daily starting the day prior to *Salmonella* challenge (5 × 10^7^ CFU, *n* = 7 each group, at 48 h p.i.). Bacterial burdens in the liver **(E)**, spleen **(F)**, MLN **(G)**, cecum **(H)**, and feces **(I)** were determined, respectively. All data are shown as mean ± SEM. Student’s *t*-test was performed. Statistical significance is indicated by *p < 0.05, **p < 0.01.

Inflammasome activation and the release of the pro-inflammatory cytokine interleukin (IL)-18 in the epithelial compartment contribute to repair and restitution of ulcerated epithelium (33, 34) and host protection against mucosal bacterial infections (1, 35, 36). To determine whether there is a difference in inflammasome activation that can explain the higher susceptibility of MLKL^−/−^ mice to *Salmonella* invasion, we first evaluated caspase-1, the best characterized protein to date, which is cleaved and activated upon recruitment to the multiprotein complex inflammasome (37). Our results revealed that caspase-1 cleavage markedly decreased in the ceca of infected MLKL^−/−^ mice relative to the levels observed in the ceca of infected WT mice, whereas the expression of ASC did not appear significantly different on 6 or 48 h p.i. (Figures 7A,B). Specially, we found that GSDMD (38, 39), an executor of pyroptosis and inflammasome-dependent cytokines release, also prominently reduced in the ceca of infected MLKL^−/−^ mice (Figures 7A,B). Importantly, the amount of inflammasome-dependent IL-18 was relatively lower on 6 h p.i. (*p* = 0.1302) and significantly inhibited on 48 h p.i. in the cecum tissues of MLKL^−/−^ mice than in those of WT mice (Figures 7C,D). These results demonstrate that MLKL deficiency potently inhibits inflammasome activation during mucosal *Salmonella* infection. Subsequently, to determine whether MLKL-mediated inflammasome activation is implicated in protection against *Salmonella* infection, we treated MLKL^−/−^ mice with recombinant IL-18 (rIL-18) before *Salmonella* infection and examined whether exogenous IL-18 administration could rescue the defect in pathogen control. We found that injection of recombinant IL-18 strongly reduced the *Salmonella* burdens in MLKL^−/−^ mice, as indicated by the levels of viable bacteria in liver, spleen, MLN, cecum and feces of MLKL^−/−^ mice, and which were returned to almost WT mice levels (Figures 7E–I). In conclusion, these data establish that MLKL mediates protection against *Salmonella* epithelial localization through promoting inflammasome activation.

## Discussion

As a key component of necroptosis signaling pathway, MLKL is the most known terminal protein in this kinase cascade (14, 40). Although MLKL-mediated necroptosis is involved in host defense to microbial infection (41), the biological implications of MLKL in bacterially triggered colitis have not been characterized. Using a colitis model, we found that MLKL^−/−^ mice were more sensitive to *Salmonella*-induced colitis, as indicated by increased mortality, body weight loss, and intestinal-associated pathologic changes including cecal weight loss, goblet cells loss, submucosal edema, PMN infiltration, complete destruction of epithelial integrity, and pro-inflammatory mediator production. After gavage administration, *Salmonella* initially breaches the intestinal epithelium and then disseminates from the GI tract to colonize systemic sites, ultimately resulting in typhoid-like systemic illness (5, 9, 42). MLKL deficiency resulted in increased bacterial burdens in both intestinal and systemic sites, highlighting the key role for the MLKL in constraining *Salmonella* initial intestinal invasion and subsequent systemic dissemination.

On account of *Salmonella* is primarily a food-borne pathogen, and the primary and essential step in *Salmonella* infection is to cross the intestinal epithelial barrier (9). To better define how MLKL prevents *Salmonella* pathogenesis, it is worthwhile to investigate whether intestinal barrier function is impaired in MLKL^−/−^ mice. The intestinal mucus provides the first line of defense for the host against various infectious agent and effectively prevents enteric pathogens from reaching and damaging the epithelium (43). Our present studies recall earlier studies with mucosal *Salmonella* infection, the level of mucins synthesis in WT mice was relatively increased during infection, potentially promoting host defense by removing bacteria from the mucosal surface. However, mucin expressions were dramatically reduced in MLKL^−/−^ mice following *Salmonella* infection. Especially, mucin 2, a predominant component of the intestinal mucus layer, was also materially reduced in MLKL^−/−^ mice.

Aside from the mucins secretion, there are other factors that determine host susceptibility to an enteric bacterial pathogen. Tight junction dysfunction has been linked to a variety of local and systemic diseases (44), such as Crohn’s disease (45), leaky diarrhea (46), inflammatory bowel diseases, and inflammatory bowel disease associated colorectal cancer (47). Importantly, *Salmonella* can cause tight junction disruption in infected epithelial cells, leading to increased epithelial permeability (8, 29, 48). Thus, we hypothesize that the susceptibility of the MLKL^−/−^ mice to *Salmonella* reflects not only their impaired mucins secretion but also the exaggerated tight junction dysfunction. As expected, following infection, the expression of claudin 3 was dramatically reduced in MLKL^−/−^ epithelium. Simultaneously, *in vivo* permeabilization assay using FITC-dextran showed that fluorescence intensity of serum was significantly increased in MLKL^−/−^ mice, suggesting that intestinal barrier function is more readily disrupted in the absence of MLKL signaling.

Cell turnover is maintained by a balance between the rate of cell proliferation and apoptosis and has been offered to account for maintenance of the epithelial barrier (49, 50). Furthermore, previous reports have shown that pathologically induced epithelial cell apoptosis was associated with increased epithelial permeability (51, 52). Similarly, despite we observed that there were no significant difference in PCNA-positive cells between WT and MLKL^−/−^ mice, MLKL^−/−^ mice presented with notably increased TUNEL-positive cells during *Salmonella* infection, indicating that activation of the MLKL signal may induce a compensatory tissue homeostatic response in order to preserve the integrity of the epithelial layer during *Salmonella* infection.

Although these data suggest MLKL promoting intestinal mucosal barrier integrity following *Salmonella* challenge, we cannot exclude the possibility that the disrupted gut mucosal barrier in MLKL^−/−^ mice on 48 h p.i. is a consequence rather than the cause of increased bacteria localization. Therefore, to further determine whether the fragile intestinal barrier is a fatal defect in MLKL^−/−^ mice, we analyzed the initial interplay between *Salmonella* and the host’s gut mucosa. At 6 h p.i., intestinal pathological features of infected MLKL^−/−^ and WT mice were not obvious. The synthesis or secretion of mucins before and after acute infection was not affected in the absence of MLKL. Exhilaratingly, we observed higher *Salmonella* burdens MLKL^−/−^ epithelium at the acute stage of infection. There results suggest that MLKL potentially limits early epithelial colonization of *Salmonella* to prevent subsequent intestinal barrier disruption and bacterial systemic dissemination.

The question arose as to how MLKL limits early intestinal colonization of *Salmonella*. Using immunohistochemistry of cecum samples, we observed that active MLKL was primarily located in the crypt epithelial cells. Moreover, MLKL expression in non-hematopoietic cells had a critical role in protection during *Salmonella* infection, as evidenced by WT and MLKL^−/−^ chimeric mice. Thus, MLKL expression in IECs mediates protection against early bacterial colonization. Since the enterocyte inflammasome activation emerges as an important aspect of epithelial defense against microbial infiltration (32, 36, 53). Thus, we examined whether the inflammasome modulates MLKL-mediated intestinal protection. Strikingly, we found that *Salmonella*-induced inflammasome activation was notably inhibited in MLKL^−/−^ mice compared to WT mice. Importantly, inflammasome dependent IL-18 production in MLKL^−/−^ epithelium was lower compared with the control, and systemic administration of recombinant IL-18 strongly increased the protective effects of *Salmonella*-infected MLKL^−/−^ mice reflected by reduced bacteria colonization. This is consistent with previous data showing that IL-18 is associated with repair and restitution of ulcerated epithelium and contribute to host protection against mucosal bacterial infections (35, 54, 55). IL-1β, another inflammasome-mediated cytokine, increased in the cecum tissues of infected MLKL^−/−^ mice. Intestinal levels of IL-1β correlate well with severity of intestinal inflammation and NLRC4 inflammasome deficiency still results in IL-1β production, suggesting other signaling rather than NLRC4 inflammasome plays dominant role in IL-1β production (56). IL-1α/β and IL-18 are not required for epithelial restriction of early *Salmonella* infection (18 h p.i.), and IL-18 contributes to host protection at later stages (36 h p.i.) (32). Consistent with this finding, we observed rIL-18 restricts *Salmonella* translocation at 48 h p.i. However, MLKL^−/−^ epithelium had a modest higher bacterial burden at the acute stage of infection (6 h p.i.); it is probable that pyroptosis rather than IL-18 contributes to MLKL-mediated protection against early *Salmonella* epithelial colonization.

Recent studies have shown that necroptotic stimuli-mediated MLKL signaling can promote NLRP3 inflammasome activation in macrophages (17, 57). NLRP3 inflammasome is required for host control of mucosal pathogen *Citrobacter rodentium* (54), and *Salmonella* can also induce NLRP3 inflammasome activation in macrophages (58, 59). However, neither ASC nor NLRP3 is essential for host defense against *Salmonella* intestinal infection (60). To date, NLRP1, NLRP3, NLRC4, AIM2, and pyrin are well established to assemble a canonical inflammasome complex. Of these, actually only NLRC4 inflammasome has been shown to mediate mucosal protection against Salmonella infection (32, 56, 61). Epithelium-intrinsic NLRC4 inflammasome drives infected enterocyte expulsion to restrict *Salmonella* replication in the intestinal mucosa via activation of caspase-1 and -8 (32, 61). Therefore, we suspect that non-hematopoietic MLKL-mediated host protection may primarily rely on NLRC4 inflammasome activation, which need extensive study.

## Ethics Statement

All animal studies were conducted according to experimental practices and standards approved by the Animal Welfare and Research Ethics Committee at Jilin University (No. 20150601).

## Author Contributions

SXY, WC, and YJY designed experiments. SXY, WC, ZZL, FHZ, SQY, GQH, XXQ, JZ, KM, CTD, and JMG performed the experiments and analyzed the data. SXY wrote the manuscript. XMD, YWH, and YJY revised the manuscript. All authors read and approved the final manuscript.

## Conflict of Interest Statement

The authors declare that the research was conducted in the absence of any commercial or financial relationships that could be construed as a potential conflict of interest.

## Abbreviations

MLKL, mixed lineage kinase-like protein; GSDMD, gasdermin D; *Salmonella, Salmonella enterica* serovar typhimurium; MLNs, mesenteric lymph nodes; IECs, intestinal epithelial cells; PRR, pattern recognition receptors; TLRs, toll-like receptors; RIPK1, receptor interacting protein kinase 1; RIPK3, receptor interacting protein kinase 3; CFU, colony-forming unit; H&E, hematoxylin and eosin; PMN, polymorphonuclear leukocyte; ZO-1, zonula occludens-1; rIL-18, recombinant IL-18.
