# Supplementary material for: Non-Hematopoietic MLKL Protects Against Salmonella Mucosal Infection by Enhancing Inflammasome Activation
Source: Front Immunol. 2018 Feb 2;9:119. doi: 10.3389/fimmu.2018.00119 (PMC5801401; doi:10.3389/fimmu.2018.00119)
Supplement: Supplementary file 1 [file Data_Sheet_1.docx]

**Non-hematopoietic MLKL protects against *Salmonella* mucosal infection by enhancing inflammasome activation**

Shui-Xing Yu^#^, Wei Chen^#^, Zhen-Zhen Liu, Feng-Hua Zhou, Shi-Qing Yan, Gui-Qiu Hu, Xiao-Xia Qin, Jie Zhang, Ke Ma, Chong-Tao Du, Jing-Min Gu, Xu-Ming Deng, Wen-Yu Han and Yong-Jun Yang*

**Supplemental material**

**Figure S1**

**A**


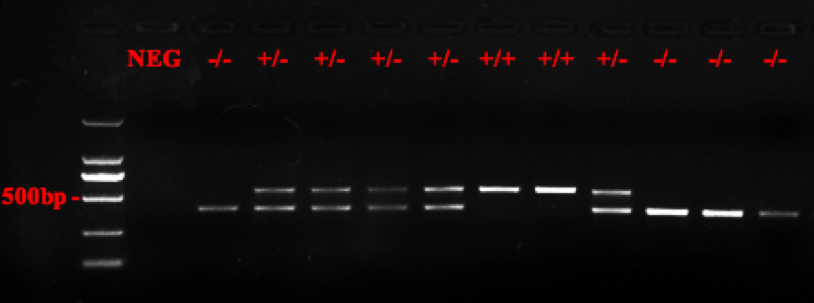


**B**


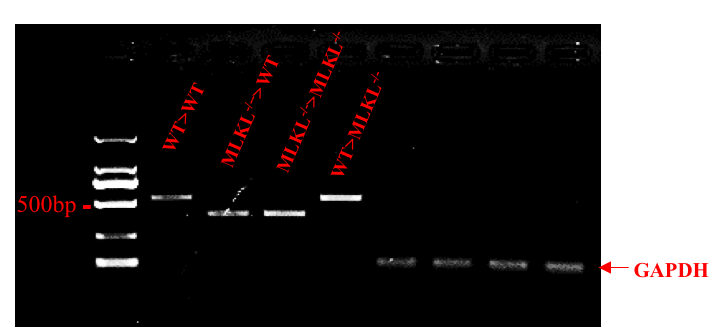


**Figure S1. Depletion of circulating cells and reconstitution with donor cells was confirmed in chimeric mice by PCR.** (A) DNA extracted from the tail of mice, and analyzed by PCR. A set of two primers that amplify sequence either from the wild type (600 bp), MLKL knockout (450 bp) or heterozygote (600 bp and 450 bp) allele. (B) DNA was extracted from splenocytes in bone marrow chimeric mice, and analyzed by PCR. Representative PCR results were shown. The following primers were used for specific amplification: 5'-AGCCCAAAGAGCAGCACAAATC-3' and 5'-AAACTT -CCAAATATGGGACTTCTTG-3'; GAPDH sense: 5'-CACCCCAGCAA-GGACACTGAGCAAG-3', antisense 5'-GGGGGTCTGGGATGGAAATTGTGAG-3'.

**Figure S2**


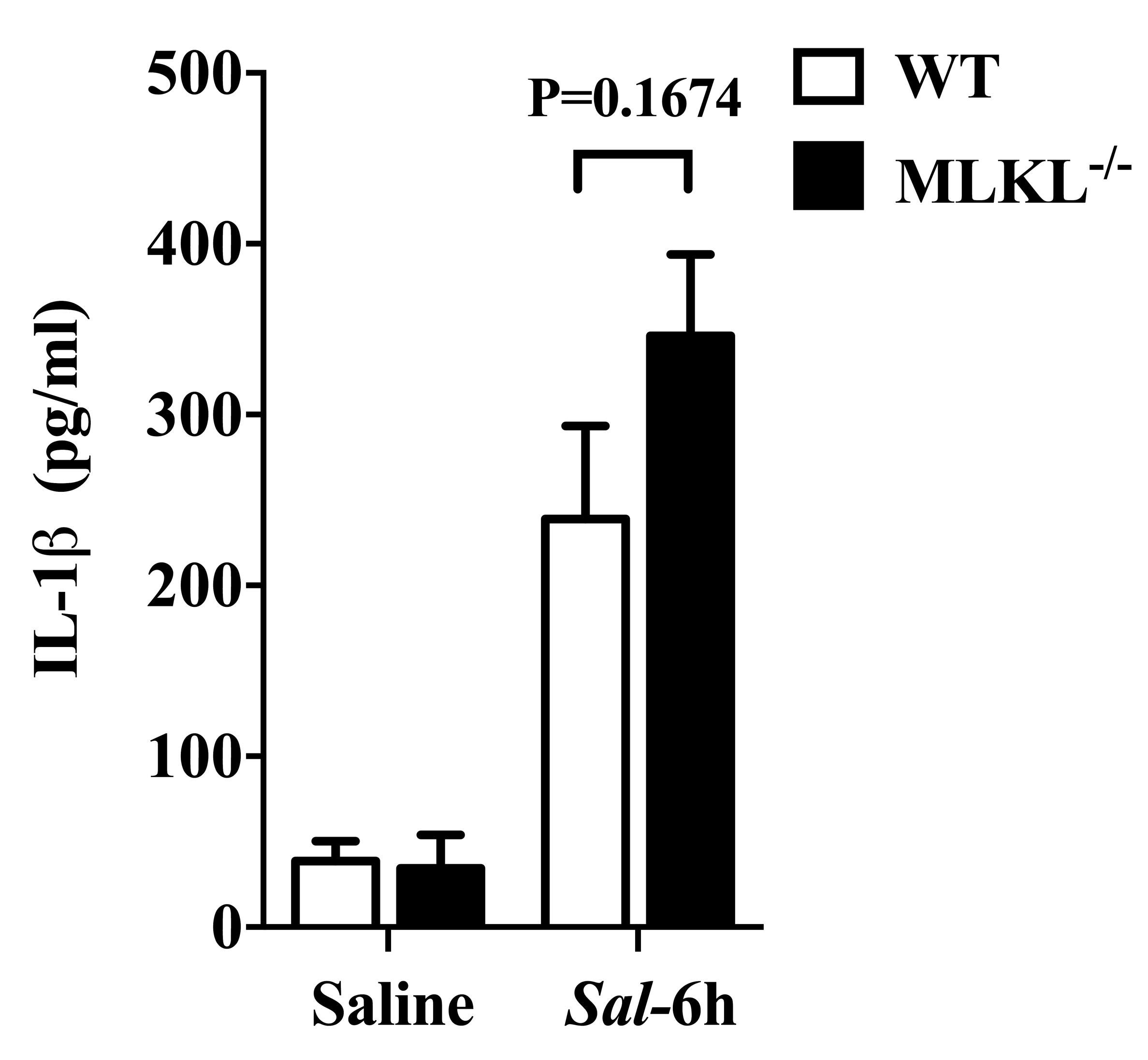


**Figure S2.** Streptomycin-pretreated WT and MLKL^-/-^ mice were orally infected with *Salmonella* (5x10^7^ CFU, n=10 each group) for 6 h. The homogenate supernatant of the cecum tissue was analyzed for the amount of IL-1β using ELISA.
